# Employment, Social Networks and Undocumented Migrants: The Employer Perspective

**DOI:** 10.1177/0038038514532039

**Published:** 2015-02

**Authors:** Alice Bloch, Sonia McKay

**Affiliations:** University of Manchester, UK; London Metropolitan University, UK

**Keywords:** employment, ethnic enclave businesses, migrant entrepreneurs, recruitment, social networks, undocumented migrants

## Abstract

This article draws on data from qualitative interviews with ethnic enclave and ethnic economy business entrepreneurs from Chinese, Bangladeshi and Turkish-speaking communities in London. Routes into business and worker recruitment practices are explored, demonstrating the centrality of social capital in the form of family and other social networks within these processes. The article investigates what employers consider the desirable characteristics of workers: trust, kinship, gender, social networks, language compatibility and the needs of the business intersect with racialised notions of workers’ strengths and characteristics. Finally, we consider changing practices in relation to the employment of undocumented migrants, in the context of an increasingly punitive legislative regime. The complex and variable impact of policy alongside the ways in which other obligations and positions outweigh the fear and risks of sanctions associated with non-compliance is revealed.

There is an increasing body of research and scholarship that explores the experiences of undocumented migrants within the labour market. Undocumented migrants – that is migrants without any legal permission to be in the country of residence – are often restricted to the most precarious jobs within the least regulated parts of the economy (Bloch, 2013; Goldring et al., 2009). Recruitment practices are often informal, with businesses and workers relying on micro social networks; consequently workers can be rendered disposable, interchangeable and sometimes subjected to poor terms and conditions of work and low pay (Cobb et al., 2009, Lockyer and Scholarios, 2004; MacKenzie and Forde, 2009; Wills et al., 2010). Although undocumented migrant workers are particularly vulnerable to poor conditions, experiences are not uniform; cleavages based on gender, ethnicity, immigration status and length of time in the country of residence intersect with experiences (Holmes, 2007; MacKenzie and Forde, 2009; Vertovec, 2007; Wills et al., 2010).

Compared to undocumented migrant workers, relatively little is known about migrant employers who own the types of businesses that are associated with informal employment practices and represent the kinds of sites where undocumented migrants and semi-compliant workers, such as asylum seekers working in breach of their asylum claim or students working longer hours than stipulated in their visas, can seek and find work (Lucas and Mansfield, 2010; Ruhs and Anderson, 2006). This article builds on the limited literature by focussing on the recruitment and employment strategies of business owners within the ethnic enclave economy. The term ‘ethnic enclave’ derives from the North American sociological literature and refers to enterprises that serve their own ethnic market and/or the general population. Their basic characteristic is that the enterprise is minority ethnic owned and that a significant proportion of the labour force is composed of migrant workers from the same ethnic group (Portes, 1981: 290–1). In using this term, we are not implying that it is only applicable to minority ethnic groups, as majority ethnic populations exhibit similar clustering. However, for minority ethnic businesses the range of operation may be more constricted in terms of available resources, including labour (Light et al., 1994; Portes, 1981; Portes and Bach, 1985).

Ethnic enclave businesses are presented in the literature dichotomously as either positive or negative, although the reality is much more complex. On the positive side, the labour market segmentation of workers within co-ethnically concentrated sectors can provide employment opportunities for migrant workers (Fong and Shen, 2010) and job creation for entrepreneurs (Wahlbeck, 2007), especially where they experience blocked mobility as a consequence of labour market discrimination and exclusion. Setting up a business can increase earning potential and represents a natural progression based on skills acquisition (Chaudhry and Crick, 2004; Kitching et al., 2009; Wahlbeck, 2007). The majority of ethnic minority led businesses are micro businesses concentrated in catering and textiles within what Kitching et al. describe as ‘… competitive, low-value-added ethnic niche sectors … [with] … low entry barriers regarding finance and skills, rendering many operators insecure’ (2009: 692–3).

Ethnic enclave businesses rely on the effective use of social capital derived from kinship networks to finance businesses, for unpaid labour provided by family members and for information, advice and encouragement (Valdez, 2008), although access to resources for business set up is gendered. Migrant women have less access to the resources necessary for business set up and are therefore less likely to be entrepreneurs (Fiscal Policy Institute, 2012). Part of the explanation is that women are more rooted within kinship networks while men have access to diverse social networks, including work based linkages, that offer wider sources of information and advice for business set up and maintenance (Renzulli et al., 2000). While among men setting up business and self-employment is seen as a good employment option, for women it can provide the opportunity to accommodate work and family life (Baycan-Levent and Nijkamp, 2011).

Networks are important, not just for setting up businesses but also for staffing because they facilitate informal recruitment within local labour markets, especially local migrant networks, offering low cost labour (Chaudhry and Crick, 2004; Kitching et al., 2009; Lucas and Mansfield, 2010). Wider co-ethnic networks, rather than just kinship based networks, can be crucial because they enable access to not only a wider pool of labour but also more diverse markets and clients. Smallbone et al. note the importance, for South Asian business owners, of connecting with local community networks ‘… in identifying the business opportunity, and in recruiting staff with the necessary language skills and cultural knowledge’ (2010: 183). However, using co-ethnic labour sourced through networks has other benefits from the employer perspective, including worker flexibility, a pool of available workers, trust and perceptions of reliability (Collins, 2002; Jones et al., 2006; Walhbeck, 2007). Social processes are seen to be efficient when hiring workers because bosses learn something about their potential employees, reducing risk (Waldinger and Lichter, 2003).

Co-ethnic workers are thought to possess desirable characteristics of which the solidarity and trust assumed by shared ethnicity features highly (Collins, 2002). Wahlbeck notes that the ‘… ethnic economy’, at closer scrutiny, seems to be an ‘economy of trust’ (2007: 552). Using informal networks for recruitment means that businesses go back to the same pool for workers and that is why, argue Waldinger and Lichter, that ‘a labor force organized around some particular social category (gender, nativity, ethnicity, geographic origin) tends to be reproduced time and again’ (2003: 219). The reproduction of ethnicity within an enclave business is not just about intra ethnic obligation. Employers make rational economic calculations and ‘group members … can be squeezed that much harder’ (2003: 155).

Within certain types of jobs, attributes and behaviour, including worker compliance, can outweigh other factors in recruitment. Anderson and Ruhs maintain that ‘… certain types of bodies (most obviously, gendered, racialized, and aged) are considered more suitable for certain types of work’ (2010: 20), especially within the most informal and least regulated parts of the workplace. Similarly, in their analysis of ethnic minority niche employment and gender, Schrover et al. observe that ‘Employers see women and immigrants as workers who are “willing”, “fit”, “able” or “suited” to do badly paid and little valued work’ (2007: 534). Such stereotypes creep into recruitment processes; the key words used to explain the recruitment of migrant workers by employers are ‘motivated, reliable, committed, excellent attitude, hardworking, flexible, and superior work ethic’ (Lucas and Mansfield, 2010: 171).

However, for migrant workers, especially for those without documents, the arrangement can be reciprocal though not always equal. Workers in these businesses, especially undocumented migrants, rely on the informal economy for livelihoods and this dependency can be the context that frames work relations (see Bloch, 2013). For those workers without the language skills of the country of residence, enclave businesses offer a compatible linguistic environment where there is no premium on English and can be the main route to employment on arrival (Ryan et al., 2008). While this might offer short-term opportunities, in the longer term the hindrance to language acquisition means that the enclave can trap workers and prevent the development of social networks seen as crucial for progression (Elrick and Lewandowska, 2008; Lancee, 2012; Nakhaie et al., 2009; Portes and Bach, 1985).

While social capital accumulation is often portrayed as vital for labour market opportunities and integration more generally, there are limitations especially for workers who remain within the ethnic enclave as a consequence of their irregular status. These ethnically bound networks are not always privileged as corridors to opportunity; as Ryan (2011) notes, bonding with people ‘like us’ is much more complex and nuanced and, specifically in relation to migration, bonding and bridging capital ‘may be too sharply constructed’. While we concur with the general position advanced by both Ryan (2011) and Anthias (2007), that ethnic-specific social networks are not necessarily positive for migrants and that a concept of the homogeneous migrant group disregards differences by class, gender and generation, our research suggests that where constraints are imposed by lack of legal status, ethnic networks may be the only available option, even where they only offer minimal or even no access to resources or information (Bloch and McKay, 2013). Thus, Anthias’s concept of mobilisability may be more useful in relation to the undocumented, as the inability of the undocumented to translate their social networks into growing resources does not constitute social capital or, as Anthias puts it, ‘social capital ethnic bonds and networks that are poorly valued in society and which do not translate easily into forms of advantage’ (2007: 792).

For employers though, workers with limited language skills and restricted options as a consequence of status offer a cheap and disposable labour supply based on ‘autocratic social relations’ (Ram et al., 2000: 44). Thus, the enclave with its pool of low paid and available workers traps these workers in a co-ethnic and mono-linguistic environment, limiting their access to social networks outside of the immediate ethnic community and preventing structural embeddedness into the wider society (Bloch, 2013; Portes, 1981; Wills et al., 2010). As a consequence, argue Jones and Ram, ethnic enclave businesses in marginal sectors of the economy survive through the ‘sweated exploitation of a captive co-ethnic labour force, people effectively excluded from alternative opportunities in the mainstream labour market’ (2010: 164).

This article examines the experiences, perceptions and practices of employers who own a business or businesses within the ethnic enclave or the ethnic economy. We focus on four main areas: the reasons for forming a business; preferred worker characteristics; recruitment practices; and employment practices in relation to undocumented migrants within the context of an increasingly punitive immigration regime. Prior to exploring the empirical data, the research approach and the sample are elaborated on.

## The Research Approach

Qualitative interviews with 24 employers from Bangladeshi, Chinese and Turkish speaking communities (including Kurds from Turkey and Northern Cypriots) in London, carried out as part of a larger project *Undocumented Migrants, Ethnic Enclaves and Networks: Opportunities, Traps or Class based Constructs?* funded by the Economic and Social Research Council, form the empirical basis of this article. The three groups were selected for their sizeable presence among London’s minority ethnic communities but also their migration histories, reasons for migration and pathways to the UK have been different, providing the variance we were looking for in the study. The employer interviews formed part of a larger project that included in-depth interviews with 55 undocumented migrant workers from the same groups. This article is concerned with the employers’ perspectives given the relative paucity of research and scholarship in this area; elsewhere we focus on undocumented migrants’ experiences of the labour market (e.g. Bloch and McKay, 2013).

Employers were identified for interview using chain referral methods starting at multiple access points for greater sample heterogeneity (Bloch, 2007; Penrod et al., 2003). Initial points of access included cold calling at businesses, gatekeepers from community organisations, the networks of community researchers who carried out interviews with undocumented migrants and in so doing made connections with employers, and community stakeholders who helped negotiate access to employers. Employers are difficult to access, especially those who might be employing undocumented migrants or contravening other official employment regulations (Ram et al., 2000). Difficulties of access were accentuated by the sensitive nature of the research. Penrod et al. note that, when trying to access the field, ‘researchers must be culturally sensitive to perceiving and respecting the risks and sensitivities of the targeted research group’ (2003: 104). Our success at finding employers willing to be interviewed was due in part to the timing of the fieldwork, which took place after most of the interviews with undocumented migrants had been carried out and so we were able to effectively utilise some of the networks that had been developed.

**Figure 1.**
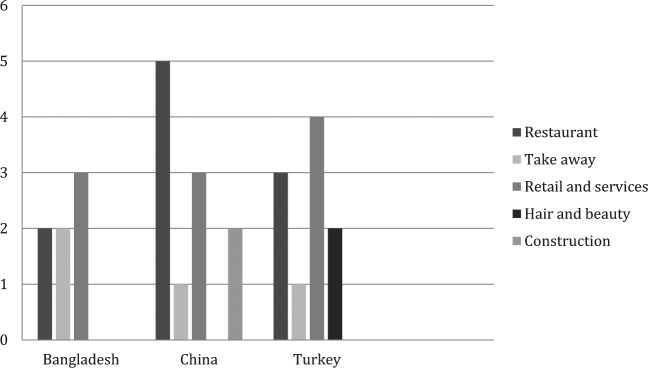
Current business by country of origin group. *Note*: Retail and services includes shops, food import/export, car garage and a Chinese medicine provider. Base number: 24 employers and 28 types of business

The university-based research team carried out all interviews with employers, anonymised the transcripts of potential identifiers and changed the names of all interviewees and those referred to during the course of the interviews to protect individuals from identification. In three cases one of the community researchers, who had acted as a gatekeeper, also attended the interview at the request of the interviewee and in two interviews, where the interviewee had difficulty in translating a word or phrase into English, she provided minimal interpreter assistance. We are aware of the potential corruption of research data where transmitted through interpreters (Bassnet, 1994; Temple and Moran, 2006) but do not believe that this was a risk in relation to these interviews, which were primarily conducted in English. The final sample of employers comprised seven Bangladeshi, eight Chinese and nine Turkish entrepreneurs of whom six were Kurds from Turkey, two were Turkish and one was from northern Cyprus. Five interviewees were female and 19 were male. With the exception of one Bangladeshi heritage woman who ran a family owned business, all the other employers interviewed were migrants born outside of Britain. Length of time in Britain ranged from nine years to over 40 years and a small minority (four) had been asylum seekers in the past. Figure 1 shows the type of business; where entrepreneurs owned businesses in different sectors each sector is coded.

Restaurants and take-away shops accounted for half of all businesses. The businesses varied in terms of their clients and markets; some catered almost exclusively to co-ethnic customers, notably funeral arrangements and culturally specific clothing, while others sought a more diverse customer base outside of the co-ethnic group, though also remaining within ethnic clusters, and so displayed features of ‘mixed embededdeness’ (Barrett et al., 2001; Kloosterman et al., 1999). Being embedded within migrant social networks as well as the structures of the country of residence allows migrant businesses to access both markets and can be highly advantageous economically (Kloosterman et al., 1999).

## Initial Routes into Business

Pathways into business were variable; some came from families with pre-existing businesses or had networks of family friends where experience was gained as a precursor to self-employment, others took opportunities when they arose and some had experienced blocked mobility, glass ceilings and discrimination. Light et al. described the ethnic economy as ‘a school for entrepreneurs’ (1994: 72) and certainly our study revealed evidence of this. Mr Uddin, for example, arrived in the UK from Bangladesh at the age of 10; he talks about working in restaurants owned by his uncle before opening his own business:

I understood the business very quickly, trained very quickly, became waiter, manager, I could run his business … I was only nineteen after two/three years working for him with the help of him and the help of my father I opened my first restaurant.

Other businesses emerged through opportunities, including a funeral business co-directed by a Bangladeshi woman who was the only British born interviewee. In the following quote she describes how and why her grandfather set up the business:

He was a very religious man and because of his religious ideals when his friend dies … he realised that there was no one there to Islamically bathe him and to do the preparation according to the Qur’an so he asked to do it and also asked that if anybody else ever came to them to call on him and he would do all the things. And then he got name just in the very small community because it was so small it went by word of mouth that he dealt with anyone that died. (Ms Ali)

Blocked mobility was a motivation for setting up business; Mr Rahman came to the UK as a student from Bangladesh in 1986, was unable to find a job in his profession (electronics) so instead worked in the restaurant sector. Finding a job in catering was easy as most of the restaurant owners were from Bangladesh and, for him, opening his own take-away once he had learned the business seemed like a natural progression. Similarly, Mr Mahmood, who had worked as an accountant mainly for small co-ethnically owned garment factories, set up a shop in the ethnic enclave selling culturally specific clothing because he was ‘… not getting a good job’.

Operationalising different forms of capital for business start-up were evident and linked closely with family networks. Family members or a combination of family members and savings mostly financed new business ventures; very few had used banks or other lenders. Among our female entrepreneurs, one had inherited a stake in the family business while the others had all gone into business with their husband, reflecting the gendered patterns of entrepreneurship and social networks (Renzulli et al., 2000). Chaudhry and Crick (2004) noted a pattern among smaller Chinese restaurants of the husband responsible for the kitchen and food ordering and the wife and children running the front of the house by taking and delivering the orders. Such a division of labour was the basis on which Ms Zhou, a Chinese interviewee whose first business was a take-away shop with her husband, structured their enterprise. Trust and reliability gained through family and other social networks were crucial to decision-making about employment and these qualities influenced the desired characteristics of employees, as the next section explores.

## Current Business: Preferred Worker Characteristics

A pattern of informality within recruitment processes was found among the majority of businesses, though two of the larger businesses – a Chinese medicine provider and a food import/export business – had human resource departments. However, even these large businesses relied on co-ethnic employees. In the case of the Chinese medicine provider it was due to the preference for Chinese trained and experienced practitioners, while the import/export business initially preferred Turkish-speaking workers due to language. Even though the current workforce had diversified, there was a majority of Turkish workers:

… if I employ a British, probably the word I’m using he’s not going to understand everything … That’s why we’re trying to choose who can speak our language and speak English as well … we have from all nations but still the majority is still Turkish. (Mr Simsek)

Relying on co-ethnic workers sometimes reflected the needs of the business, especially the customer base or specialist skills such as chefs. In other cases there were clear perceptions of who was suitable for the work, based on ethnic stereotypes of workplace commitment, ability and attitudes. In short, ‘good’ workers, from the employer perspective, often equated with workers from the same ethnic group. Within restaurant and take-away businesses, the role within the business was crucial. Migrant workers tend to work ‘back-of-house’ as cooks and kitchen porters and have little or no contact with customers (Baum et al., 2007). Working with customers meant English was usually a requirement; employers were usually more flexible about who was employed ‘front of the house’. Having a chef from the country of origin of the cuisine was seen as a basic requirement:

I can’t have an Englishman as a creative chef [if it’s someone from home] … I don’t have to explain to him … how it was done back home. (Mr Hasan, Bangladesh)

Similarly, the need to have a Chinese chef was made by an owner of a Chinese restaurant:

In the restaurant [you] cannot employ any other people … It’s ok for waiter, waitress, not in the kitchen. They got to know how to cook the Chinese food. (Mr Chen)

Kitchen porter work, which is notoriously hard with long hours and low pay, carried with it an assumption that it was most suitable for co-ethnic workers who were prepared and equipped to do such demanding work. According to Mr Hasan, ‘Bangladeshi people are very hard working, physically hard working.’ Similarly, Chinese employers held the same perception of and preference for Chinese workers:

… there’s only so many jobs that you can give to non-Chinese-speaking people … and experience has shown that they tend not to be as reliable. I’m not being racist here I’m just telling you my experience … they just don’t turn up … so we don’t actually employ a lot of non-Chinese. (Mr Peng)

Waldinger and Lichter found similar racialised notions of workers for the most menial and lowest paid jobs, as employers thought they ‘were more likely to come with the desired traits’ (2003: 159). These traits were what they perceived to be a differential work ethic between ethnic groups and a greater compliance among non-white workers who were willing to work for less. Stereotypes operate within different groups with employers identifying workers from their own country of origin as ‘good’ workers. In the case of the Bangladeshi restaurants, homogeneity extended to gender – employees were almost exclusively male. When talking about workers, the male pronoun was mostly used and so a ‘good’ worker equates with a Bangladeshi male.

Businesses geographically situated and embedded within the ethnic enclave specified that the ability to speak the community language and/or understand customs and culture was a job prerequisite. Mr Mahmood, the owner of a clothing shop, for example placed greater emphasis on Sylheti than English because ‘… ninety-five percent of the customers are from the Sylhet’. Similarly, the owner of a gadget and electronic repair shop with a customer base mainly from the local Chinese community emphasised the importance of community languages rather than English. These businesses were less able to exploit the weak ties of different networks to access wider markets due to the geography of the business in the heart of an ethnic enclave, the reliance on co-ethnic workers and, in the case of the clothing shop, the cultural specificity of the goods on sale. For such businesses expansion to more diverse markets is difficult and workers and markets remain largely homogenous (Smallbone et al., 2010).

Business owners were, not surprisingly, very cautious about whom they put in management roles. Some of the entrepreneurs, with smaller or single business concerns, managed the day-to-day affairs of the business themselves, while those with shops that were open 24 hours a day and/or with multiple businesses put either family members or people with whom there was a long and established working relationship in management roles. Family was central to businesses, as was the idea of family, although this was not necessarily based on ties of biological kinship but instead on the ties of trust, or on other forms of shared experience such as geography or politics. In the following quote Mr Sindi from Turkey, who owned a supermarket, talks about the running of the shop at night and a worker who has been with them for many years including in a previous business:

Even when we’re doing the tile business, he was working for us *he is like our brother*, we are always together. We leave everything to him at nights and go home and sleep. (Mr Sindi)

Family took on a different and gendered meaning for our female Bangladeshi heritage co-director. For her, the issue was not only about trust, but working with family also enabled her, as a Muslim woman, to be able to work in an environment where she would otherwise be excluded:

… as a Muslim woman, to work with a group of men is quite difficult but it isn’t compromised here, he’s my uncle so I’m very calm and relaxed here, my father’s also here and my cousin, so I – I’m void of many normal situations that some people would find themselves in, that me as a Muslim woman would find difficult working in an office with lots of men. (Ms Ali)

The next section explores the ways in which businesses recruit their workers, showing the importance of informal co-ethnic networks.

## Recruitment Practices

Differences in recruitment practices depended on either the size of the individual business or the number of businesses owned. Where the business entrepreneur owned one business and worked there most days they tended to control recruitment and often used informal processes. Those with larger businesses or multiple business concerns gave either human resources or managers the responsibility of hiring staff. In restaurants, chefs participated in the process of hiring kitchen staff. The main ways in which workers were found was through the social networks of either the employer or his or her workers. However, a minority of businesses advertised, usually in minority newspapers, and so targeted people from the same ethnic and/or linguistic group. Employers also experienced cold callers and had different ways of dealing with these requests. Agencies were rarely used, but where they were they tended to be non-statutory and often run by people from the same ethnic group. In this section we explore the ways in which businesses recruited their workers, noting the primacy of the informal processes derived from social networks across all three groups.

In the following quote, a Bangladeshi restaurant owner describes the functioning of diaspora based networks in worker recruitment:

… the Bangladeshi community … it’s a very small community … go back, er, fifteen years/twenty years you had ninety-five percent of the resident came from that district of Sylhet … So they knew each other from school and college so they still kept in touch … So when a friend opened a restaurant, you know, it would be ‘oh, I’m opening a restaurant’ and the other friend would go and help … if somebody wanted a job they phoned … and somebody would come up and say ‘hey, so and so’s looking for a waiter, so and so’s looking for a …’. Or, the same token if you were looking for a staff for your restaurant you’d ring somebody up: ‘oh, do you of anybody free’, a waiter or a kitchen porter or a chef … (Mr Ahmed)

Turkish and Chinese employers also used social networks for recruitment. Mr Rami, a Kurd from Turkey, spent two years working in his relatives’ business in London before setting up his own off-licence in a town in the East of England where he had one employer who was a friend whom he recruited, ‘because he couldn’t find a job in London, I take [him] with me to work’. These networks are an important component of both employer and worker social capital and can take time and effort to develop. Workers want to stay in jobs, become indispensable employees and to recommend their friends when vacancies arise, while employers require hard working and reliable workers (Gomberg-Muñoz, 2010). Trust takes time and networks evolve, as the owner of an interiors firm explains:

I advertise … in Chinese [newspaper] … And that’s all to begin with. And then they’re introducing their friends and since then you don’t have to [advertise], just call: ‘hey, can you get somebody to help?’ and they – they know each other and they bring them over, so easy. (Mr Zue, China)

Using social networks in this way also has the advantage of a kind of reference, not formal, but one based on personal recommendations as one restaurant owner observes:

So we mostly rely on, erm, personal references. You know, got a friend or some relatives. (Mr Peng, China)

Using informal procedures also means workers can be found quickly to cover for unexpected gaps in staffing and/or on a temporary basis to cover illness or holidays. In the following quote a take-away shop owner is talking about how he finds workers to fill in for short periods at short notice:

Most of the time, er, friend and family, ask the family people ‘do you know anybody to work here … we need some people’. (Mr Rahman, Bangladesh)

Using social networks over agencies was preferred due to the negative appraisals of workers sent by agencies, especially statutory ones where workers were viewed as unmotivated and where there is no pre-existing social connection to the workers to act as a reference. Talking about agencies, Mr Ahmed, a restaurant owner from Bangladesh says:

Is useless. One is the people they will send are they’re not into – they’re not interested to work, they’re just following the commitment that they have to do so, so basically you’re wasting your time ringing them up and saying ‘look, send somebody’ … word of mouth and friends and recommendation, that I don’t find still in the agencies.

Agencies were not seen as providing specialist workers, and this was alluded to by one of the restaurateurs:

But I still rely on the quality of like chefs and things like that, word of mouth and friends and recommendation that I don’t find still in the agencies. (Mr Uddin, Bangladesh)

A few of the businesses carried out targeted advertising because they had specific skill requirements. The Chinese health centre, as noted earlier, preferred Chinese trained practitioners and advertised in Chinese language papers:

… in the Chinese newspaper they have a section just for Chinese medicine so all the doctors if they look for new job they know where to find it. (Ms Yin)

Businesses also experienced cold callers who they would occasionally employ if they needed someone for the business. What did these informal approaches to recruitment mean in terms of the employer attitudes to checking documents and working with undocumented migrant workers, a group that relied almost exclusively on informal ways of finding work? The next part of this article explore attitudes, experiences and decision-making about the employment of undocumented migrants within the context of an increasingly punitive immigration regime.

## Employing Undocumented Migrants: Changing Regimes and Attitudes

**Figure 2.**
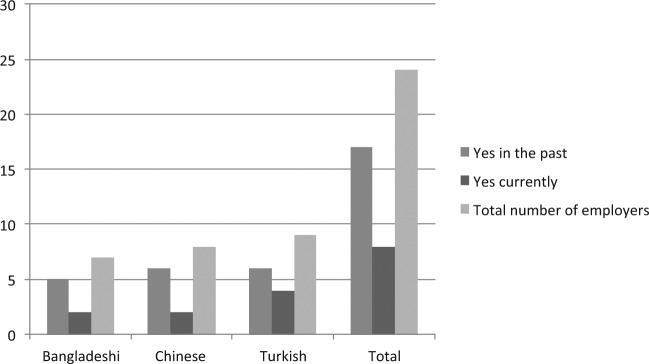
Employment of undocumented migrants in the past and currently. *Note*: Base number: 24

Most people had employed undocumented migrants in the past though fewer claimed to employ them now as Figure 2 shows. At the time of interview eight employers were open about employing irregular migrants, although 17 employers said that they did so in the past. Six restaurant or take-away shop owners, one hairdressing business and one construction company specialising in interior refurbishments said they were currently employing people without documents.

Changing employment practices were strongly linked to sanctions; employers were acutely aware of government policy. As Mr Chen from China said, ‘you’ve got to be careful these days’ and according to Mr Peng, also from China, ‘you don’t want to take any chances’. Similarly, Mr Ahmed from Bangladesh said that he doesn’t employ undocumented migrants, ‘because I can’t afford to pay that fine’. Mr Peng had Chinese restaurants and left the recruitment to the managers. Reflecting on changes he observes:

… in the old days … it was quite common to employ, [people without documents] … but since the enforcement of the law … as soon as everyone realised that that was being enforced more stringently than in the past, and yeah so we just have to adapt to the situation really. I mean these days if you turn up to a restaurant and you have no documents people just don’t want to know, certainly our ones anyway, that’s the number one thing we ask for.

Even though there were risks when employing undocumented migrants some employers were still open about their practices and their reasons. One of the take-away shop owners, who generally recruited workers with documents, used informal networks to appoint undocumented migrants when he had short-term gaps to fill:

I have taken somebody on for a couple of weeks where … a kitchen porters left ‘… oh I’ve got a cousin of mine but he hasn’t got any *card or nothing*’. ‘Well look, just send him down and while I’m sort of searching for somebody at least he get two weeks work, a bit of pocket money’. (Mr Hasan, Bangladesh)

Others presented a caring and philanthropic narrative, as the following quote from Ms Jaf, a Kurd from Turkey, illustrates:

… one example, er, like sitting in restaurant, someone walked in, really so fragile, asking for a job, I don’t need no one, he leaves the place, I am like ‘he needed a job and he couldn’t even speak English’. I run behind him, ok, Afghani guy, no paper at all, nothing, and he work with me almost seven months.

There was also an element of empathy and co-ethnic solidarity among the restaurant owners who were Kurds from Turkey:

Some people come, I mean they haven’t got papers but they are good people, they need money. They need it, if you don’t give them job what are they going to do. (Ms Tovi)

I’ve been a Kurd in Turkey, I was one of the others and I know how it feels being one of the others and now I’m giving a chance to support my people, why not? (Ms Jaf)

The other main driver for employing undocumented migrants was the requirement of kinship and community. Mr Sindi, a business owner from Turkey, talks about employing undocumented migrants:

When I first opened … I employed three undocumented people for one year but I have to, one of them was my brother in law the other one is a friend of a friend and they asked us to employ him. How I can say no? And I took the risk.

While some talked openly about their own obligations others talked about the practices of other business owners. It is possible, even likely, that they faced similar commitments but were more comfortable talking about the practices of others rather than their own, given the sensitivity of the subject. Mr Raham from Bangladesh, for example, noted that ‘… about eighty percent of the illegal immigrant is working within their relative’s businesses’. According to Mr Mahmood, government policy has resulted in a contraction of the wider employment networks but not of the obligation to family members without documents. Although approached two or three times a week about jobs by other Bangladeshi people trying to assist new arrivals or other undocumented migrants needing work, Mr Mahmood had a policy of only employing his own relatives when in need. In the quote below, Mr Mahmood talks more generally about policy:

… policy hugely affects within the catering because … they are not taking any illegal immigrants … ten years before, fifteen years before, I ring one of my friends, a restaurant owner, said that somebody came from Bangladesh, my relative. Yes, send him, send him. Now he will not accept. Maybe his own brother, own nephews, own – but at the moment, it’s very tight.

Among some employers there was haziness on the margins of employment compliance. As noted earlier, undocumented migrants can and do provide flexible labour utilised to cope with temporary labour shortages or for short periods of time while they are ‘sorting out their documents’. There were also contradictions and ambiguities within some individual narratives. In the following interview extracts, Mr Zue, who had a construction company and so worked in different locations, presents differing accounts of his practices during the course of his interview:

… my wife won’t allow me to employ them with no documents.

I don’t mind sometimes because my location is changing all the time so I’m taking a risk … right now … we only have two [with] no documents.

I dare not to employ them because you’ve got to understand the five thousand pounds per person. And also you are involved with the court case and trials.

… some of the Chinese people from mainland, they are really rely on job to support the family, otherwise they won’t come all the way from China, six thousand mile, and that’s the problem. Because of the immigration law and they’re a bit scared but they’re still taking a risk, same me and same them, we both.

The mobility of the jobs within the construction business meant that this employer was taking a calculated risk. Similarly, some Chinese workers in our study, including Li who was 42 and had been in Britain for eight years, reported a preference for construction jobs rather than restaurant work due to the mobility of the work place:

I had worked in catering before … But I thought it was too risky to work in the kitchen, because you could be caught anytime … There you would have to go in and out the same place everyday. You would be easily spotted and caught [by the police]. It’s not like working on the building sites, where you move about from one place to another. (Li from China)

Other employers, though aware of the sanctions, were nevertheless undeterred. Mr Serhati, a Kurd from Turkey, owned a restaurant and had been raided by the police in the past. Although he asked people for their status when they approached him for work this was merely a formality and did not preclude their employment. On some occasions he had also employed people and later found out that they were undocumented. While some employers said they gave a few weeks’ notice in these instances, he simply stated that ‘I just take the risk’. Having been an asylum seeker he was sympathetic to the predicament of those without papers who were excluded from the regular labour market.

The narratives of employers in this research differs from that found by Jones et al., who note that the informal nature of employment means that among employers, ‘the recruitment of illegals is a passive and largely accidental process’ (2006: 143). Our research suggests that employers are generally aware of whom they employ and have reasons for their strategy based on economics, obligations, political positions, ethnic and/or political solidarity and humanity. The more punitive policy climate has created an acute awareness of the consequences of breaches and therefore greater caution among employers. However, the research also suggests that there are powerful factors within ethnic enclave and ethnic economy businesses that outweigh the potential consequences of breaches and sustain employment practices. While fewer said they were now employing undocumented migrants compared to in the past, the data strongly suggest that government intervention will not change practices, because some ties and political positions outweigh the risks posed by government policy.

## Conclusion

This article shows that social capital in the form of family, friends and wider co-ethnic community networks is crucial to the development and running of businesses operating within the ethnic enclave economy. These businesses are largely functioning outside of commercial loan structures and outside of formal recruitment practices and instead rely on family and social networks for funding, staffing and worker recruitment.

Among the business owners we interviewed there was a preference for co-ethnic workers in certain roles or as a result of the location and/or nature of the business. One reason was the specialist skills and the linked perceptions around authenticity. Chefs and Chinese health practitioners trained and experienced in the country of origin were considered a requirement for the job. Among businesses within ethnic enclaves providing services or goods to members of the same ethnic group, the relevant community language was crucial. In addition to the needs of the business, employers also expressed racialised and sometimes gendered notions of what constituted a ‘good’ employee, as well as preferences for a more homogenous labour force with the linguistic and cultural understandings and assumptions that accompany it.

While previous research suggests that low cost and flexibility motivate the utilisation of new migrant and undocumented migrant workers, our employers for the most part did not present these as influential factors in their employment practices. Instead, their focus was mostly on the practical needs of the business and on the perceived – albeit racialised and gendered – attributes of the workers in relation to those needs. Unsurprisingly, employers presented themselves in a positive light, often describing themselves as ‘good’ employers, offering fair wages and providing an almost paternalistic level of support to their workers should they encounter difficulties in their lives outside of the workplace. In some of the businesses, workers have been there for a number of years and trust was a key component of these working relationships. This trust was derived from the ties of kinship, recommendations from others from the same ethnic group network and the longevity of some working relationships.

Different employment practices were presented with regard to the recruitment and retention of undocumented migrant workers. All employers were aware of the potential risks and the implications on their businesses of large fines, especially given the micro nature of some of the businesses operating in saturated and low return sectors. The reality, however, is that employment practices are complex and variable and the impact of policy, in relation to the employment of undocumented migrants, is capricious and inconclusive.

Our research suggests that there is not one single analysis of ethnic enclave business formation, worker recruitment strategies and employment practices. Instead, a range of circumstances influence practices relating to social networks, kinship, community, political obligations, ethnic and political solidarity, immigration status, availability of workers, linguistic, cultural and gender preferences, perceptions of ‘good’ workers, government policy and of course economic considerations. Crucial to any analysis is the awareness and understanding of fluidity. Economic and policy contexts change and with these changes so too do business practices. Moreover, within the wider economic and political contexts, ethnic enclave businesses operate within communities and the social capital derived from these networks remains the frame in which businesses operate.
